# Cases of Epilepsy

**Published:** 1854-07

**Authors:** Charles H. Baker

**Affiliations:** Buffalo


ART. IV.—*Cases of Epilepsy.* Reported to the Buffalo Medical Association, by CHARLES H. BAKER, M. D.

In the treatment of no disease is more uncertainty manifest than epilepsy. Almost every medical man has his favorite remedy, and yet it is but giving utterance to the experience of all physicians to say that cases, apparently hopeless, have been cured, while often the most sanguine anticipations of recovery have been disappointed.

The idea of specifics, in the management of disease, has been sadly rebuked in the presence of morbid nervous conditions.

The mere enumeration of remedies proposed for the cure of epilepsy would almost answer as an index to a treatise on materia medica; while the discarded list would be scarcely less valuable. It may safely be inferred that epilepsy dependent upon organic disease is incurable, except as the organic disease is removed. Doubtless it is equally safe to infer that it is curable when dependent upon mere functional derangement or sympathetic action. A more through knowledge of its pathology is needed to insure our prognosis from error, and render our treatment less empirical. How far nux vomica, or its active principle strychnine, may be found to control or modify epileptic disease, is yet problematical. That in many cases it does have a powerful influence is certain. Several that have come under my notice have been treated with the tinct. nux vomica with the most happy results.

My first trial of this remedy was in 1850, on a young man aged 19, who had been afflicted with epilepsy since 1843, (seven years.) The disease was supposed to have been induced by a severe injury in the lumbar vertebra, and one of his hips, from the kick of a horse, as the injury was followed very shortly by a fit.

After about two months he had a second one more severe than the first. From that time he was subject to frequent attacks at irregular intervals. Generally—within the first four or five years—as often as every three or four weeks.

The longest period of intermission was two months. Latterly the fits had increased in frequency and severity, and at the time I saw him were occurring every second and third day; occasionally two or three in one day.

Almost any unusual excitement was liable to bring on a paroxysm. The insensibility which invariably followed the convulsions generally lasted from two to six hours. In one instance, however, it held him eighteen hours. A somewhat singular phenomenon, in his case, was the exhibition of a voracious appetite, on the return of consciousness. And unless guarded he would completely gorge the stomach. When once satisfied, his appetite would be moderate.

Between the fits, the secretion of tears was so abundant as to give him the appearance of weeping. His friends had noticed a subsidence of this secretion, generally three or four hours preceding an attack. This was the only premonitory symptom they had observed. His physical system had evidently been retarded in its development, not only in size but also in virility. His appearance would not indicate an age above 14. The countenance was dull and downcast, with an almost idiotic expression of the eye. His gait shuffling and unsteady. His intellect, never remarkably bright, was apparently nearly destroyed, and whatever knowledge he had acquired from books was entirely lost. He was entrusted with no kind of business, as he had neither memory nor perception to enable him to take care of himself. There was, withal, a general costive habit of the bowels, with occasional complaints of headache.

A full dose of comp. cath. pill was directed to be administered at once, and to take the tinct. of nux vomica prepared after the following formula:

℞ Rasp'd. nux vomica, ʒi.

Proof spts., ℥iv. M.

Dose—20 drops before meals, three times daily. He was put on a milk and vegetable diet, and ordered to be kept free as possible from all excitement. The bowels in the mean time to be kept open with laxatives.

Learning some ten days subsequently that there had been no recurrence of the fits, the tinct. was discontinued until the usual premonitory symptom, (the drying of the eyes, before mentioned,) showed itself. Then to recommence and continue the same dose and frequency for two or three days, when the medicine was to be laid aside until further called for. Under this treatment he continued entirely free from fits, his general appearance improveing and strength increasing. His mental faculties also seeming to brighten so that in the spring of 1851 he commenced laboring on the farm, performing light work, running errands, &c., with apparent benefit to his health.

In the winter of 1851-52 I lost sight of him, having removed from that vicinity.

A letter dated 8th May, 1852, from Dr. F. T. Bishop, who resides in his neighborhood, informed me that the patient had taken, to that date, ℥iv. of the tincture, that he yet continued free from his old disease, that during the past winter he had made some progress in his studies, and was increasing in strength and ability to labor.

Having been so long free from his malady he became careless of himself, and as I subsequently learned, was, during the summer, more ambitious than prudent. In September of that year, after an unusually hard day's work in the harvest field, he had a fit, but lighter than formerly. He immediately quit work and recommenced the use of the medicine, of which he had taken none for several weeks. After a few days he resumed his ordinary labor with no repetition of the convulsions.

During the past year he has, I understand, been to work by the month in Canada, considering himself perfectly cured.*

A singular feature in this case, is the facility with which the symptoms yielded to treatment. During the seven years he had suffered, he had frequently been under medical treatment, but without any appreciable benefit, so

*Since this was written I have learned that the young man has had another fit this spring, the result of gross carelessness and recklessness on his part. This makes two fits he has had since he commenced the use of the tinct. It should be added here, that he has taken none of the medicine for over a year.

far as checking the force or frequency of the fits. Not the least interesting item is, the improvement, mental and physical, which has followed the subsidence of the convulsions.

A brother of the patient remarked to me a few days since—that I should hardly be able to recognize him—the change had been so great. He had grown from a puny appearing lad weighing less than 100, to a full sized man of 160.

Why should the growth and development of the body have been retarded? Has the epileptic diathesis any, and if so, what effect on the function of nutrition?

My second trial of this remedy was in 1851, in the case of a female who had been subject to fits previous to puberty, but which had left her at that time for some five years, when they were reproduced by a dysmenorrhœa, the result of a cold, during a catamenial period.

She had been suffering since then nearly ten years. There was no regular periodicity in the accession of the convulsions. Sometimes but one, occasionally three or four fits in the course of a month. The most marked effect of the disease, in her case, was a tendency to moroseness and irritability of temper. With her there was a general costive habit of the bowels. Is not costiveness of bowels an almost constant attendant upon diseases of this character?

The tincture nux vomica in 20 drop doses, three times daily, was prescribed, with occasional laxatives. After having commenced the use of the medicine she enjoyed a perfect immunity from the fits nearly two years.

Within that time she had married, became pregnant, attempted to produce abortion by means of oil tansy. Gastritis supervened, but she succeeded in destroying the fœtus and her own life.

In another case of a female, the fits had occurred simultaneously with menstruation for over three years. She has had but one attack since she commenced the use of the tincture, a little more than a year since.

I am far from laying claim to nux vomica or any of its preparations as a specific in epilepsy. And yet, I doubt not it will be found to possess great value as a remedy in that protean disease.

Much less do I claim any originality in prescribing this article in epilepsy. The only approach to originality is in the strength of the preparation as used for this complaint. The formula for the tinct. in the U. S. Dispensatory, makes it eight times the strength of that used in the foregoing cases. My attention was first called to the remedy by noticing a statement of its effects in nervous headaches, in the Buffalo Medical Journal for May, 1849, page

724. Having tested its virtues in that complaint and been gratified with its admirable effects, it occurred to me that it might prove equally valuable in other functional derangements of the nervous system. The correctness of the conjecture is apparent in the cases that have come under my observation.

Should the foregoing facts induce others to test their value and importance, my object in presenting this subject to the Association will have been accomplished.

It may be proper to add, I should have no confidence in the remedy in cases arising from organic disease of the brain or spinal marrow. But in all cases where epilepsy is the result of functional disorder, especially in asthenic subjects, it will, I think, be found AS valuable a remedy as we possess for the treatment of this formidable malady.

*Buffalo, June,* 1854.

